# Evolution of HIV-1 envelope towards reduced neutralization sensitivity, as demonstrated by contemporary HIV-1 subtype B from the United States

**DOI:** 10.1371/journal.ppat.1011780

**Published:** 2023-12-06

**Authors:** Lindsay Wieczorek, Eric Sanders-Buell, Michelle Zemil, Eric Lewitus, Erin Kavusak, Jonah Heller, Sebastian Molnar, Mekhala Rao, Gabriel Smith, Meera Bose, Amy Nguyen, Adwitiya Dhungana, Katherine Okada, Kelly Parisi, Daniel Silas, Bonnie Slike, Anuradha Ganesan, Jason Okulicz, Tahaniyat Lalani, Brian K. Agan, Trevor A. Crowell, Janice Darden, Morgane Rolland, Sandhya Vasan, Julie Ake, Shelly J. Krebs, Sheila Peel, Sodsai Tovanabutra, Victoria R. Polonis

**Affiliations:** 1 U.S. Military HIV Research Program, Walter Reed Army Institute of Research, Silver Spring, Maryland, United States of America; 2 Henry M. Jackson Foundation for the Advancement of Military Medicine, Bethesda, Maryland, United States of America; 3 Infectious Disease Clinical Research Program, Department of Preventive Medicine and Biostatistics, Uniformed Services University of the Health Sciences, Bethesda, Maryland, United States of America; 4 Walter Reed National Military Medical Center, Bethesda, Maryland, United States of America; 5 Brooke Army Medical Center, Fort Sam Houston, San Antonio, Texas, United States of America; 6 Naval Medical Center Portsmouth, Portsmouth, Virginia, United States of America; 7 Diagnostics and Countermeasures Branch, Walter Reed Army Institute of Research, Silver Spring, Maryland, United States of America; National Institute for Communicable Diseases, SOUTH AFRICA

## Abstract

Subtype B HIV-1 has been the primary driver of the HIV-1 epidemic in the United States (U.S.) for over forty years and is also a prominent subtype in the Americas, Europe, Australia, the Middle East and North Africa. In this study, the neutralization profiles of contemporary subtype B Envs from the U.S. were assessed to characterize changes in neutralization sensitivities over time. We generated a panel of 30 contemporary pseudoviruses (PSVs) and demonstrated continued diversification of subtype B Env from the 1980s up to 2018. Neutralization sensitivities of the contemporary subtype B PSVs were characterized using 31 neutralizing antibodies (NAbs) and were compared with strains from earlier in the HIV-1 pandemic. A significant reduction in Env neutralization sensitivity was observed for 27 out of 31 NAbs for the contemporary as compared to earlier-decade subtype B PSVs. A decline in neutralization sensitivity was observed across all Env domains; the NAbs that were most potent early in the pandemic suffered the greatest decline in potency over time. A meta-analysis demonstrated this trend across multiple subtypes. As HIV-1 Env diversification continues, changes in Env antigenicity and neutralization sensitivity should continue to be evaluated to inform the development of improved vaccine and antibody products to prevent and treat HIV-1.

## Author summary

HIV-1 has evolved and diversified over the last forty years of the pandemic. These changes have shifted viral properties in such a way that the effectiveness of current therapeutic HIV-specific broadly neutralizing antibodies may be reduced over time. Understanding these changes will allow for the development of improved vaccine and antibody products. In this study, we generated a panel of 30 HIV-1 strains from the United States from samples collected between 2017 and 2018. We characterized the sensitivity of these contemporary HIV-1 strains using 31 antibodies that can inhibit, or neutralize, infection and compared the results to those of viruses from earlier in the pandemic. Our results show that antibodies that were effective against HIV-1 strains from earlier in the pandemic are not as effective against current HIV-1 strains. This trend in reduced sensitivity over time is occurring globally, as similar results were observed in Thailand and Africa. These results will inform the development of more effective antibodies and vaccines to elicit them.

## Introduction

The HIV-1 pandemic has been ongoing since 1981 and has resulted in an estimated 84.2 million infections [1]. The global spread of HIV-1 occurred as distinct viral subtypes expanded into different geographical regions [2]. Group M HIV-1 is divided into nine subtypes, including A-D, F-H, J and K, multiple circulating recombinant forms (CRFs), including the prominent CRF01_AE and CRF02_AG, as well as unique recombinant forms [3]. Much of the subtype distribution of group M HIV-1 is preserved today, however diversification has continued over time [4–7].

HIV-1 subtype B is the primary driver of the HIV-1 epidemics in North and South America, Europe, Australia, the Middle East and North Africa [2]. While HIV-1 incidence rates in the United States (U.S.) have declined by as much as 73% between 1984 and 2019, an estimated 34,800 people acquire HIV-1 yearly and reduced incidence is not consistent across all populations [8]. Over the last four decades, considerable efforts have been made to develop biologic interventions to reduce HIV-1 transmission and disease progression. To date, HIV-1 vaccine candidates have not proven efficacious and have failed to elicit neutralizing antibodies effective against circulating, tier 2 HIV-1 envelope (Env) strains [9]. In the face of these challenges, passive immunization strategies for the prevention and treatment of HIV-1 are also being pursued [10,11].

Broadly neutralizing antibodies (bNAbs) can prevent HIV-1 acquisition through several mechanisms [12,13] and have been shown to be protective *in vivo* [14–17]. While plasma antibodies with the breadth and potency of bNAbs have been shown to develop naturally, they only occur in 20–30% of individuals after 2–3 years of living with HIV-1 [18–22]. Prophylactic and therapeutic use of synthetic antibodies may therefore prove effective in the absence of protective active immunity. However, the successful use of these bNAbs may be limited by their potency and breadth against circulating HIV-1 strains, as demonstrated by recent Antibody Mediated Prevention (AMP) trials.

Two AMP trials have evaluated the efficacy of bNAb prophylaxis on HIV-1 acquisition. HVTN 703/HPTN 081 and HVTN704/HPTN 085, were designed to evaluate the protective effect of passive immunization of the HIV-1 bNAb VRC01, which targets the Env CD4 binding site (CD4bs). VRC01 bNAb administration was found to be 75% effective at preventing infection by HIV-1 strains that were susceptible to neutralization by VRC01, with an *in vitro* sensitivity defined as IC_80_ <1 μg/ml [23]. These studies demonstrated that the *in vitro* neutralization sensitivity of the HIV-1 Env from the bNAb recipient dictated the preventative efficacy of the bNAb [23]. These results suggest that bNAbs can be effective at preventing infection when used against circulating, neutralization-sensitive HIV-1 strains and establish baseline potency for potential monoclonal antibodies (mAbs) for HIV-1 prophylaxis.

Understanding the neutralization sensitivity of circulating HIV-1 is therefore critical for prophylactic mAb and vaccine development. Since the beginning of the HIV-1 pandemic, studies have demonstrated a diversification of HIV-1 subtypes and reduction of the neutralization sensitivity of HIV-1 Env [4–7,24,25]. In this study, we sought to create a contemporary (2017–2018) panel of subtype B Envs from the U.S. and to characterize their neutralization sensitivity to mAbs. We compared neutralization sensitivity of contemporary Envs to Envs from earlier decades and demonstrated significant reductions in subtype B Env neutralization sensitivity over time. Additionally, we demonstrate that the most significant reduction in neutralization sensitivity occurred at target epitopes that were most sensitive to neutralization at the beginning of the pandemic. Results from this study could inform development of improved antibody and vaccine products to prevent and treat HIV-1 and the data strongly support the development of next-generation bNAbs for improved efficacy.

## Results

### Development of a contemporary HIV-1 subtype B Env panel

**Fig 1:**
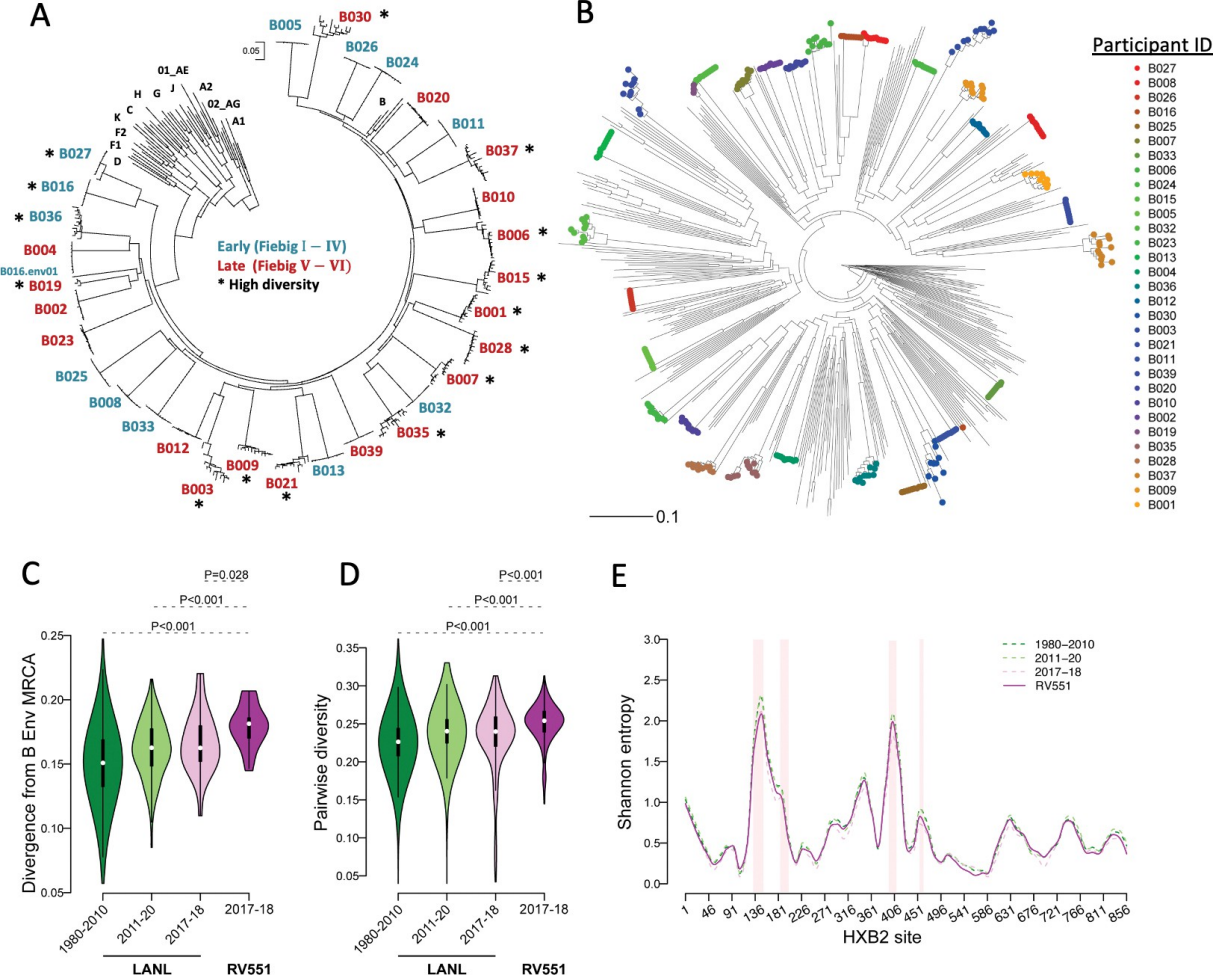
Phylogenetic relationship of contemporary U.S. subtype B Envs. (**A**) Contemporary subtype B HIV-1 Envs were cloned from post residual HIV test samples from 30 U.S. military personnel and sequence diversity was evaluated. Fiebig stage of Env isolation is shown in blue (Fiebig I-IV) and red (Fiebig V-VI); high Env diversity is marked with an asterisk. (**B**) The phylogenetic relationship was evaluated between the contemporary U.S. subtype B Envs from this study (colored symbols) and subtype B Env sequences collected between 2011–2020 in the U.S. (gray lines) downloaded from the Los Alamos National Laboratory (LANL) HIV sequence database. (**C**) Divergence from the subtype B Env amino acid most recent common ancestor (MRCA) sequence and (**D**) pairwise diversity was determined for the consensus Envs from this study (n = 32, magenta), U.S. Envs from 2017–2018 (n = 26, pink), 2011–2016 (n = 239, light green), and prior to 2011 (n = 823, dark green) in the LANL database. Significant differences were determined between consensus Envs from this study and the LANL subset by a Mann Whitney U test. (**E**) Shannon entropy was estimated at each HXB2 site for the different env alignments, shown as matched colored lines. Pink highlights represent the Env variable loops V1 –V5, from left to right.

Contemporary subtype B HIV-1 envelope genes (*envs*) were cloned from 30 post residual HIV-1 test plasma collected between 2017 and 2018 from the routine universal screening of U.S. military personnel as part of the RV551 protocol designed to surveil the molecular virology and immunobiology of HIV-1 incidence and prevalence. Twelve individuals were at early stages of infection (Fiebig I-IV) and 19 individuals were at intermediate or late stages of infection (Fiebig V-VI). We sequenced 371 *envs* from the 30 individuals and evaluated sequence diversity within and between study samples (Fig 1A). In general, individuals in Fiebig stage V or VI showed higher Env diversity than participants in Fiebig stages I-IV (Fig 1A). However, higher Env diversity was observed for two individuals with early-stage (Fiebig II) infections. Sequences from one individual, designated as B027, demonstrated the presence of multiple founder variants and another individual, B016, was determined to have a dual infection. HIV-1 Env amino acid sequences from this contemporary U.S. subtype B panel were then aligned with subtype B Env sequences available in the Los Alamos National Laboratory (LANL) HIV database that were isolated between 2011 and 2020 in the U.S. (Fig 1B). Subtype B Envs from this panel intersperse with subtype B Envs from the past decade (represented as gray lines only). These results suggest the subtype B Envs included in this panel encompass the diversity of subtype B HIV-1 Envs observed in the U.S.

### Increased sequence diversity of contemporary subtype B Env

HIV-1 Env majority consensus sequences were generated for 30 individuals. Consensus sequences were constructed separately for each of individual B016’s dual infection viruses. We calculated the divergence from the subtype B most recent common ancestor (MRCA) and the pairwise diversity for the Envs from this panel (2017–18, n = 32) and for three sets of publicly available Env sequences collected in the U.S. prior to 2011 (n = 823), between 2011–2020 (n = 266) and in 2017–2018 (n = 26). While Envs from the RV551 panel were significantly more divergent from the subtype B Env MRCA (Fig 1C) and had greater pairwise diversity (Fig 1D) than the subsets of LANL sequences, these sequences were within the expected range of divergence and diversity, i.e. the divergence and diversity values found for RV551 were nested in the distribution corresponding to the larger datasets of circulating sequences (smaller subsets of sequences taken from a set of sequences can have varying (lower or larger) point estimates).

Shannon entropy was estimated at each HXB2 site for the different env alignments. The average entropy across sites was 0.63 (hypervariable regions = 2.20) for Envs from the RV551 panel compared to 0.66 (2.40) for LANL sequences collected prior to 2011, 0.67 (2.39) between 2011–2020, and 0.59 (2.16) between 2017–2018 (Fig 1E). The variability seen among RV551 sequences corresponded to HIV-1 Env variability patterns.

### Neutralization sensitivity of subtype B HIV-1 Env pseudoviruses

Contemporary U.S. subtype B Envs from the RV551 protocol were then cloned into expression vectors. Thirty-one HIV-1 pseudovirus (PSV) stocks were produced, including one PSV from each individual; the infectious PSV with Env that was closest to the individual’s consensus sequence was then studied. PSV coreceptor usage was determined; 1 PSV utilized both the CXCR4 and CCR5 coreceptors, while the remaining 30 PSVs utilized only the CCR5 coreceptor. The CXCR4 utilizing PSV was derived from an individual at Fiebig stage IV and was excluded from further analysis.

**Fig 2:**
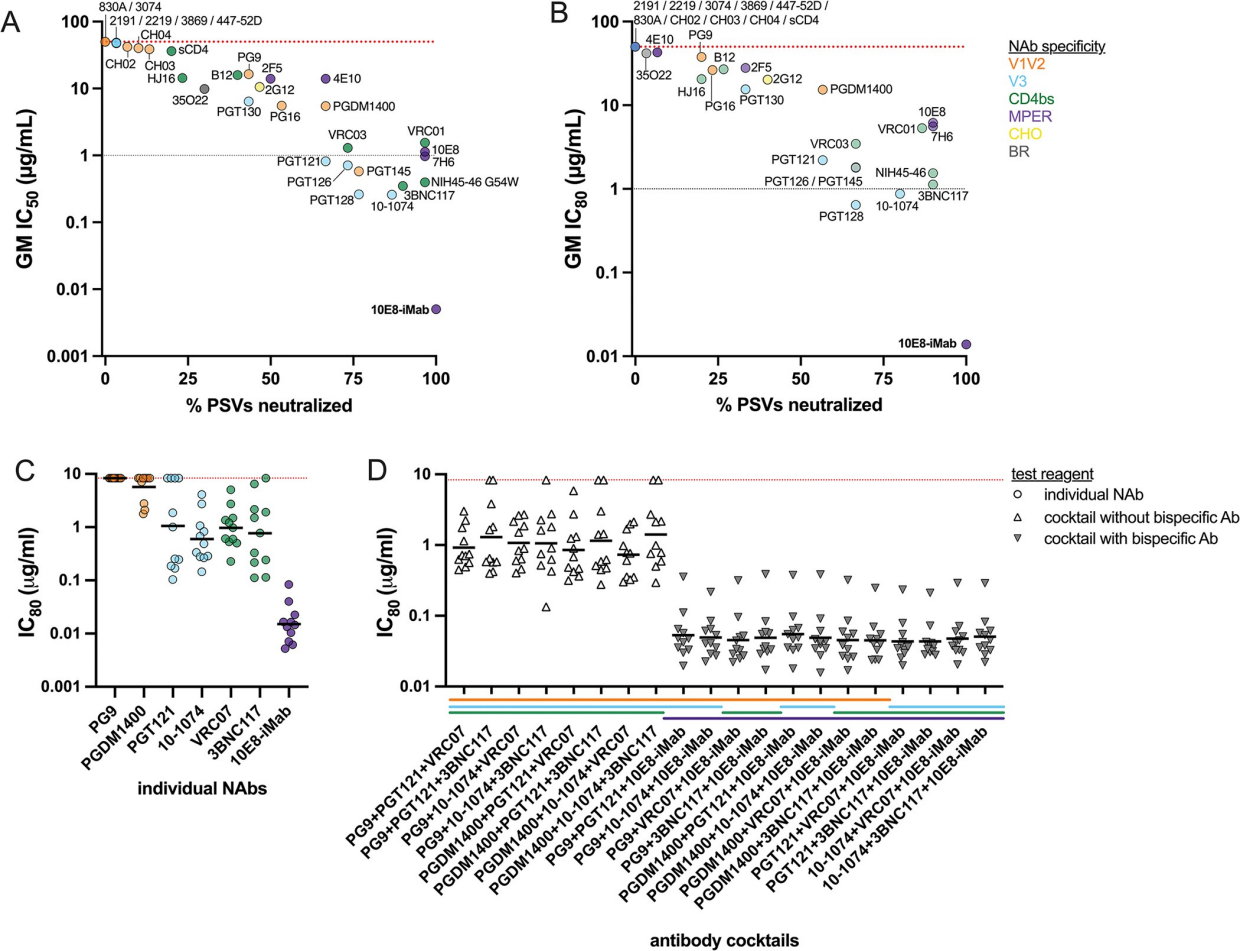
Neutralization sensitivities of contemporary subtype B HIV-1 Envs. The neutralization sensitivity of the contemporary, CCR5-utilizing, subtype B pseudoviruses (PSVs) was determined using 31 neutralizing antibodies (NAbs) or reagents. NAbs are colored by specificity, including: V1V2 loop (orange), V3 loop (blue), CD4 binding site (CD4bs, green), membrane proximal external region (MPER, purple), glycan (CHO, yellow) and bridging region (BR, gray). Geometric mean (GM) (**A**) IC_50_ and (**B**) IC_80_ scores were calculated for each NAb against all PSVs and compared with the percent of PSVs neutralized by each NAb. The red dotted line at 50 indicates the negative cutoff value for this assay; the gray dotted line at 1 indicates the estimated NAb IC_80_ value for treatment efficacy, as determined by the AMP studies. Neutralization potency of clinically relevant antibodies and antibody combinations was evaluated against early-stage, subtype B HIV-1 Envs. (**C**) NAbs were tested individually (shown as circles), or (**D**) in combination (shown as triangles; open if no bispecific antibody was present and gray if bispecific antibody was present). Combinations of NAbs included three NAbs that targeted different domains, as indicated by the x-axis title and the colored legend.

Neutralization sensitivity of each contemporary U.S. subtype B PSV was assessed using 31 neutralizing antibodies (NAbs) or reagents targeting the Env V1V2, V3, CD4 binding site (CD4bs) and membrane proximal external region (MPER) domains. To demonstrate overall sensitivity of contemporary subtype B PSVs to each NAb, we calculated geometric mean IC_50_ and IC_80_ scores and the percentage of viruses with >50% or >80% neutralization observed for each NAb (Fig 2A and 2B). Greater than 50% neutralization was observed for 29 NAbs, while greater than 80% neutralization was observed for only 21 NAbs. NAb IC_80_ values <1 μg/ml were observed for only three NAbs, V3-specific 10–1074 and PGT128 NAbs and 10E8-iMab (ibalizumab; Fig 2A and 2B). 10E8-iMab is a bispecific antibody that was engineered to target the HIV-1 Env MPER at the 10E8 contact site and the host cellular CD4 molecule [10,26]. 10E8-iMab potency against the contemporary subtype B PSVs ranged from IC_80_ of 0.0052 to 0.0834 μg/ml, with a geometric mean IC_80_ of 0.0159 μg/ml; 100% of the PSVs tested were neutralized by 10E8-iMab (Fig 2B).

We then combined three NAbs of different specificities to improve potency over the use of a single NAb. NAbs currently under clinical development [10,27–32], including V1V2 NAbs PG9 and PGDM1400, V3 NAbs PGT121 and 10–1074, CD4bs NAbs VRC07 and 3BNC117 and MPER NAb 10E8-iMab, were used to evaluate the neutralization sensitivity of early-stage contemporary subtype B PSVs against all antibody cocktail combinations at the same total starting concentration as used for testing single mAbs. The range of IC_80_s is shown for each NAb (Fig 2C). The NAb combinations with consistent neutralization potencies of IC_80_ <0.1ug/mL contained 10E8-iMab. When 10E8-iMab was used at 1/3 of the dose in the mAb cocktail, the potency slightly decreased compared to using 10E8-iMab alone.

PSV neutralization sensitivity was also compared for the contemporary Envs isolated from early-stage infection and those isolated from late-stage infection (S1 Fig). No significant difference between the geometric mean IC_50_ scores was observed between PSVs from early versus late-stage infection.

### Reduced neutralization sensitivity of subtype B HIV-1 Env over time

The neutralization sensitivities of all contemporary subtype B PSVs were compared to historic subtype B strains from prior decades. For this analysis, we utilized neutralization data for historic PSVs available in the CATNAP (Compile, Analyze and Tally Nab Panels) Server on the Los Alamos National Laboratory (LANL) HIV database. For all meta-data analysis, we utilized data from all HIV strains that matched our decade, location, and subtype selection criteria; HIV strains were removed if they were known to utilize the CXCR4 coreceptor. Limited information was available in the LANL HIV database regarding the infection stage of Env isolation or PSV neutralization sensitivity. The number of HIV strains included in all meta-data analyses are shown in S1 Table. To ensure concordance between data generated in our MHRP laboratory and previously published CATNAP data, we performed a bridging analysis using 3 PSVs and 18 NAbs and observed a highly significant correlation between IC_50_ endpoints (S2 Fig, p<0.0001, r = 0.9043).

**Fig 3:**
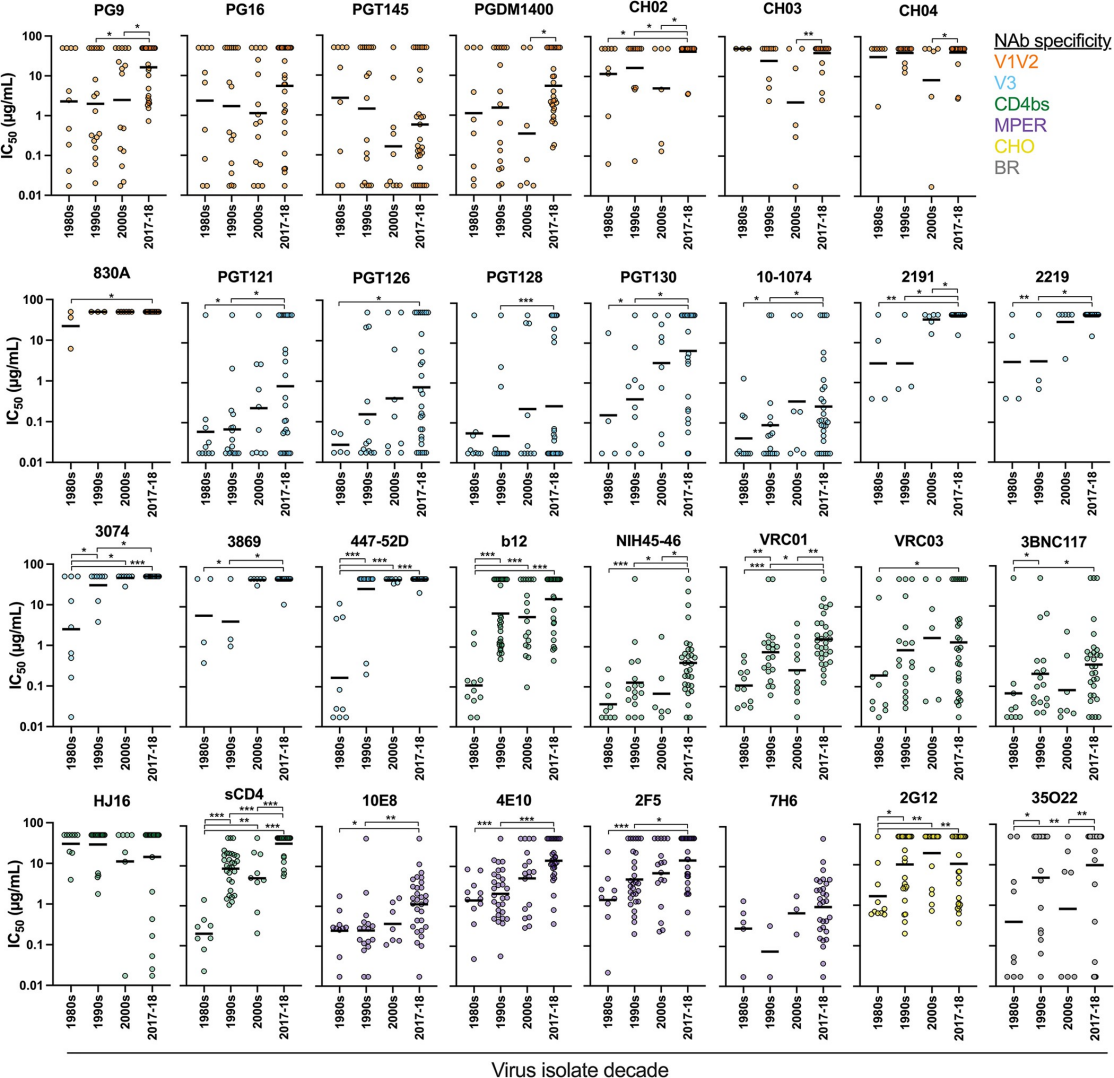
Changes in neutralization sensitivities of subtype B HIV-1 Envs over time. Neutralization sensitivities of contemporary U.S. subtype B pseudoviruses (PSVs) (2017–18) were compared with U.S. subtype B PSVs from earlier decades. Each graph demonstrates the neutralization profile of one neutralizing antibody (NAb), as indicated in the title above each graph. Symbols are colored by the NAb specificity, with V1V2-specific NAbs in orange, V3-specific NAbs in blue, CD4bs NAbs in green, MPER NAbs in purple, glycan-specific (CHO) NAbs in yellow, and bridging region-specific NAbs in gray. Significant differences were determined by Mann Whitney U test; * = p<0.01, ** = p<0.001, *** = p<0.0001.

Contemporary subtype B PSVs had significantly higher IC_50_ values than viruses from the 1980s (22/31 NAbs), 1990s (16/31 NAbs), 2000s (10/31 NAbs), as shown in Fig 3. The CATNAP HIV database had limited neutralization data available for other viruses from the 2010s. Significant differences between IC_50_ values of subtype B PSVs from different decade periods are indicated. Overall, contemporary subtype B PSVs were less sensitive to NAb neutralization than historic subtype B PSVs.

**Fig 4:**
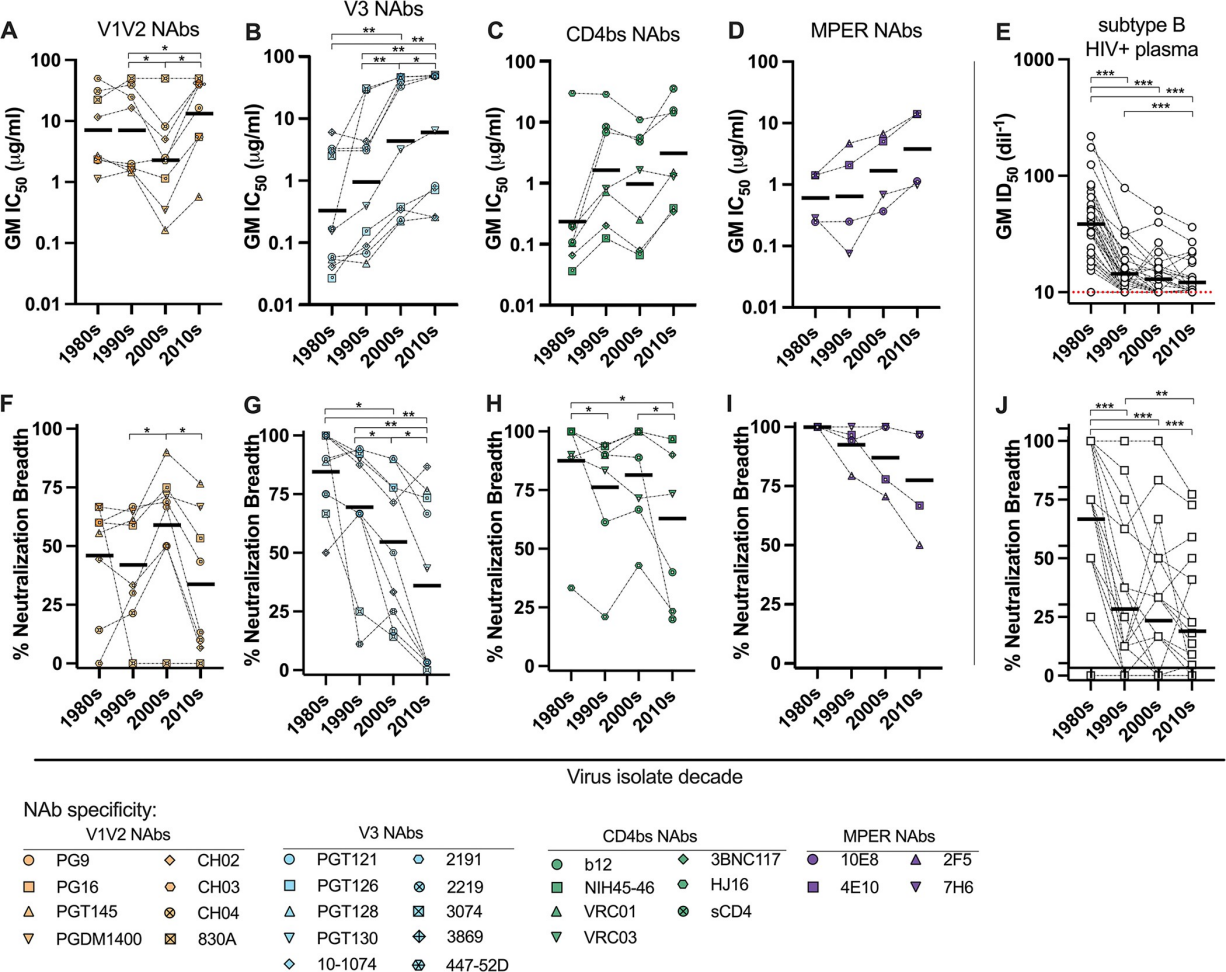
Changes in domain-specific neutralization sensitivities of subtype B HIV-1 Envs over time. For each neutralizing antibody (NAb), the geometric mean (GM) IC_50_ and percentage of pseudoviruses (PSVs) neutralized, was determined for all PSVs from each decade period, as shown in Fig 3. The trends for NAb potency (**A**-**D**) and breath (**F**-**I**) over time are shown by NAb specificity, including (**A** and **F**) V1V2 specific NAbs, (B and G) V3 NAbs, (**C** and **H**) CD4bs NAbs and (**D** and **I**) MPER NAbs. This neutralization analysis was repeated using 33 subtype B HIV+ plasma samples against PSVs from each decade period; the (**E**) GM ID_50_ and (**J**) percentage of PSVs neutralized, was determined for all PSVs from each decade. Significant differences were determined by Wilcoxon matched-pairs signed rank test; * = p<0.01, ** = p<0.001, *** = p<0.0001.

We then evaluated Env domain specific changes in subtype B PSV sensitivity over time. For each NAb, we created a geometric mean score of IC_50_ values for all PSVs from a given decade period (Fig 4A–4D). Similarly, we calculated the percentage of PSVs from a given decade period that were neutralized by each NAb (Fig 4F–4I). For this analysis, we included the contemporary panel of 2017–18 PSVs with other PSVs from the 2010s, when available. NAbs were then grouped by target Env domain and the domain trends over time are shown. We observed significant and continuously reduced neutralization sensitivity to V3 glycan NAbs over time (Fig 4B and 4G); similar trends were observed for the CD4bs and MPER targeting NAbs (Fig 4C, 4D, 4H, and 4I). While slight variations over time were observed for the V1V2 NAbs, the trend was not significantly directional (Fig 4A and 4F). Significant differences over time were determined by rank comparison, as shown, as well as by trend analysis for ordered differences. NAbs targeting the V3, CD4bs, and MPER demonstrated a significant trend in increasing order of median IC_50_s (p = 0.0001, 0.0135, and 0.0249, respectively) and decreasing order of median breadth (p = 0.0001, 0.0598, and 0.0098, respectively) over time.

We evaluated this trend of reduced Env neutralization sensitivity over time using polyclonal neutralizing reagents. We tested the neutralization potency of 33 plasma samples from individuals with subtype B HIV-1 against PSVs from the different decade periods, including 5 PSVs from the 1980s (SF162 was excluded from mean, median and statistical calculations), 8 PSVs from the 1990s, 6 PSVs from the 2000s, and 22 PSVs from the 2017–18 contemporary panel, including 11 from Fiebig I-IV and 11 from Fiebig V-VI (S3 Fig). The 11 PSVs with the highest titer were chosen for each group. We created a geometric mean ID_50_ score and determined the number of PSVs neutralized for each plasma across each PSV decade period (Fig 4E and 4J, respectively). Similar to the trends observed for the mAbs, we observed significantly decreasing PSV sensitivity (as indicated by decreasing geometric mean [GM] ID_50_, S3A Fig) to the polyclonal plasma and a decreasing order of median IC50s (p<0.0001) and breadth (p<0.0001, S3B Fig)) over time. Additionally, we stratified the HIV+ plasma samples by decade period of sample collection and observed a reduction in plasma potency and breadth over time (S3A and S3B Fig). A heat map showing all of the individual plasma reactivities is shown in S3C Fig; the SF162 data points were not included in calculations, as this represents an outlier as an exceptionally sensitive tier 1 virus.

**Fig 5:**
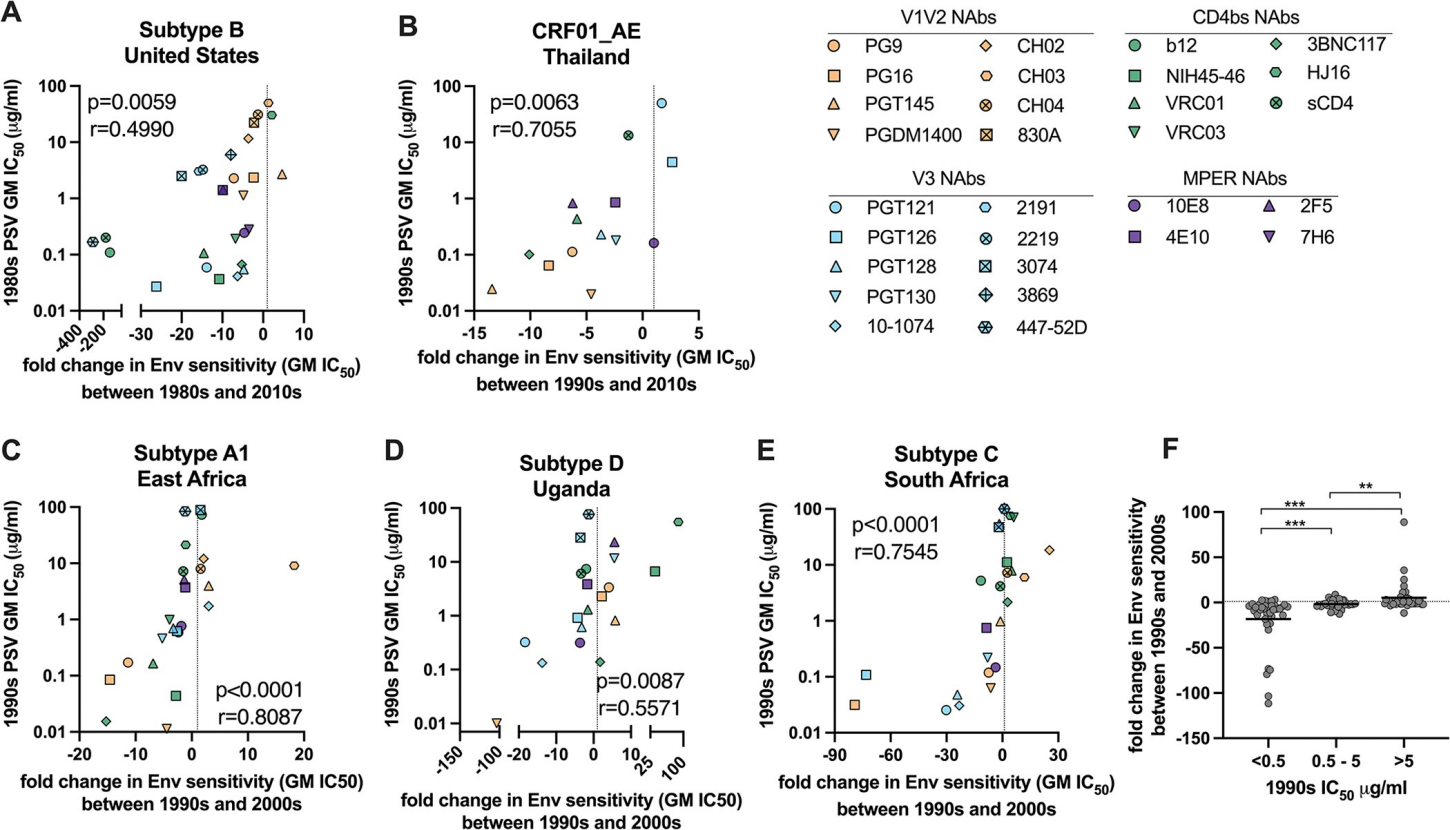
Associations between initial neutralizing antibody potency and rate of declining neutralization potency over time. For each neutralizing antibody (NAb), the geometric mean (GM) IC_50_ was determined for all PSVs from an earlier and later decade period, as shown in the x-axis title. Fold change in NAb potency was determined between the earlier and later decade periods. A positive fold change in Env sensitivity to a given NAb indicates increasing sensitivity (earlier PSV GM IC_50_ > later PSV GM IC_50_), while a negative fold indicates decreasing sensitivity over time (earlier PSV GM IC_50_ < later PSV GM IC_50_). The vertical dashed line at 1 indicates no observed change. The correlation between the fold change in NAb potency over time and the potency of the NAb against PSVs from the earlier decade period are shown for (**A**) subtype B from the United States, (**B**) CRF01_AE from Thailand, (**C**) subtype A2 from East Africa, (**D**) subtype D from Uganda, and (**E**) subtype C from South Africa. Individual NAbs are shown as unique symbols colored by target specificity, including NAbs targeting the V1V2 (orange), V3 (blue), CD4bs (green) and MPER (purple) domains. Correlations were evaluated by Spearman correlation analysis. The summary of fold change in NAb potency over a ten-year period is shown for (**F**) all NAbs from all regions; data are stratified by initial NAb potency and significant differences were determined by Mann Whitney U test; ** = p<0.001, *** = p<0.0001.

NAbs with the greatest potency against 1980s PSVs appeared to have the greatest rate of neutralization resistance over time. We therefore evaluated the fold change in NAb potency against the PSVs from the 1980s and 2010s in comparison with the initial NAb potency against 1980s PSVs (Fig 5A). A positive fold change in Env sensitivity to a given NAb indicates increasing sensitivity over time (1980s PSV IC_50_ > 2010s PSV IC_50_), while a negative fold change in Env sensitivity indicates decreasing sensitivity over time (1980s PSV IC_50_ < 2010s PSV IC_50_). The dashed vertical line on the x-axis at 1 indicates no change was observed over time. We identified a significant direct correlation between changes in U.S. subtype B Env neutralization sensitivity over time and initial 1980s Env sensitivity (Fig 5A; p = 0.0059, r = 0.4990). This indicates that NAbs with the greatest initial neutralization potency experienced the greatest longitudinal reduction in neutralization potency (increased resistance).

To determine whether this trend was applicable to other subtypes, we evaluated neutralization data from the LANL CATNAP database and our previous work [33], to characterize four additional subtypes (Fig 5B–5E). Due to the limits in data availability, not all NAbs are demonstrated and the fold change in Env sensitivity was determined over a two- or three-decade period, as indicated in each chart. Similar trends in the association between initial NAb potency and rate of potency decline over time were observed for CRF01_AE from Thailand (Fig 5B; p = 0.0063, r = 0.7055), subtype A1 from East Africa (Kenya, Rwanda, Tanzania and Uganda; Fig 5C; p<0.0001, r = 0.8087), subtype D from Uganda (Fig 5D; p = 0.0087, r = 0.5571) and subtype C from South Africa (Fig 5E; p<0.0001, r = 0.7545). Changes in NAb potency over a ten-year period (1990s to 2000s) were determined for all NAbs and regions; the results are summarized in Fig 5F. NAbs with an initial potency of <0.5 μg/ml IC_50_ demonstrated a significant reduction in potency, whereas NAbs with an initial potency of >5 μg/ml IC_50_ demonstrated a significant increase in potency over time, indicating that changes in both sensitivity and resistance were observed. Minimal change in potency was observed for NAbs with IC_50_s between 0.5–5 μg/ml. Of note, the Ugandan subtype D viruses showed a less frequent decrease in sensitivity, as compared to the other 4 subtypes.

### Subtype B Env features associated with reduced neutralization sensitivity

**Fig 6:**
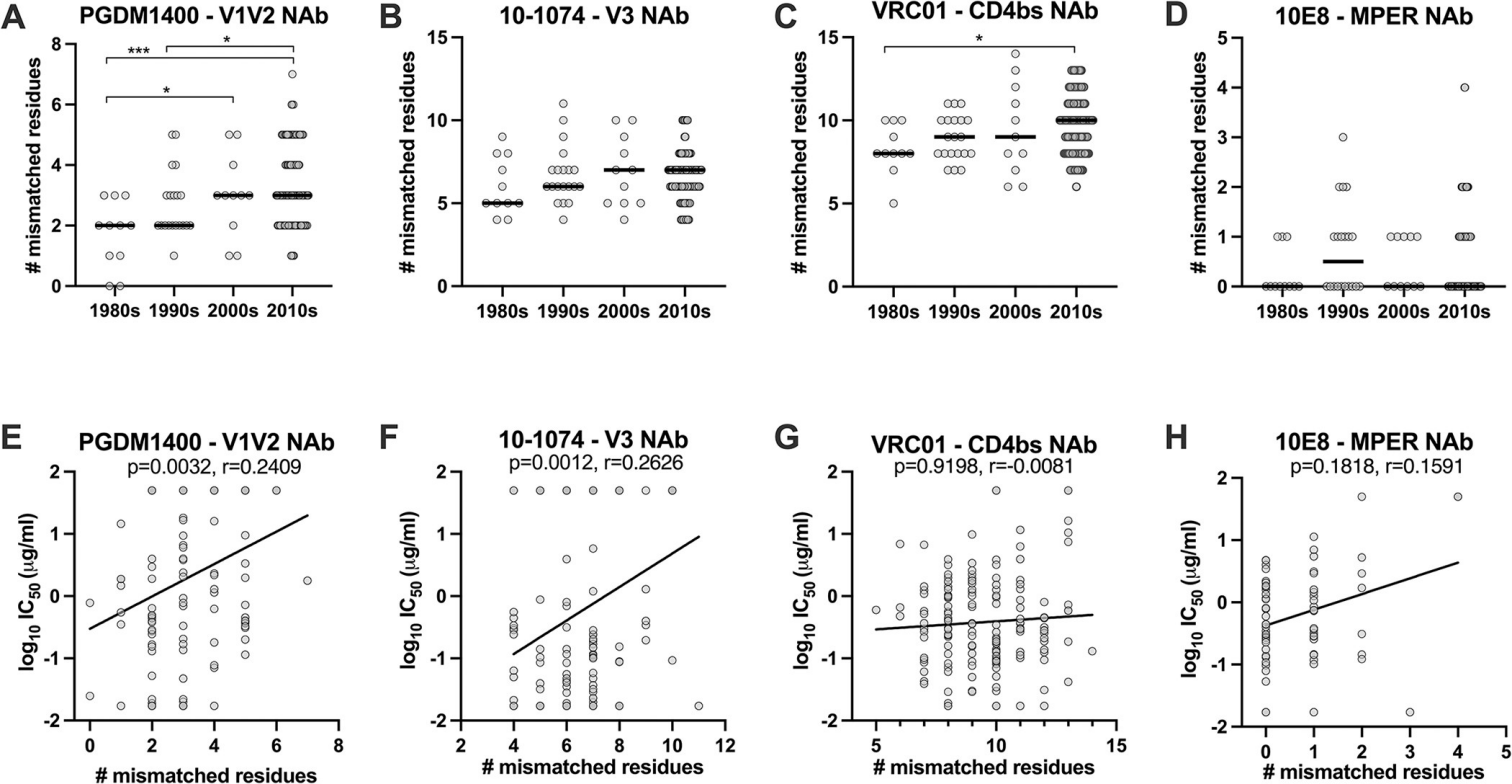
Changes in subtype B Env neutralizing antibody contact site deviations over time and impact on Env neutralization sensitivity. Subtype B Env sequences for pseudoviruses included in the neutralization analysis for each decade period were analyzed to determine the number of mismatched amino acids from the HXB2 reference strain that occurred in contact sites for neutralizing antibodies (NAbs) (**A**) PGDM1400, (**B**) 10–1074, (**C**) VRC01, and (**D**) 10E8. The relationship between the number of mismatched residues in the NAb contact site and Env NAb neutralization sensitivity were determined for (**E**) PGDM1400, (**F**) 10–1074, (**G**) VRC01, and (**H**) 10E8. Significant differences were determined by Mann Whitney U test; * = p<0.01, ** = p<0.001, *** = p<0.0001. Correlations were evaluated by Spearman correlation.

Lastly, we evaluated changes in Env features that may impact the observed reduced neutralization sensitivity. For this analysis, we used Env sequences for the PSVs used to compare neutralization sensitivity over time, shown in Fig 3. Utilizing the subtype B HXB2 strain as the reference sequence, we calculated the number of mismatched amino acids in the contact sites for four NAbs, including PGDM1400, 10–1074, VRC01 and 10E8. Envs were grouped by decade to demonstrate the accumulation of contact site mismatched amino acids over time. The number of mismatched amino acids significantly increased over time for PGDM1400 and VRC01 (Fig 6A and 6C); a trend towards increasing number of mismatched amino acids was observed for 10–1074 (Fig 6B) and no trend was observed for 10E8 (Fig 6D).

We then evaluated the relationship between the number of mismatched residues in the NAb contact site and Env neutralization sensitivity to that NAb. While the data were widely distributed, we observed a significant direct correlation between the number of mismatched residues and reduced neutralization potency for NAbs PGDM1400 (Fig 6E; p = 0.0032, r = 0.2409) and 10–1074 (Fig 6F; p = 0.0012, r = 0.2626); no significant associations were observed for NAbs VRC01 or 10E8.

To visualize potential changes in NAb contact site amino acids over time, we generated logo plots for each NAb using Env sequences downloaded from the LANL sequence database for U.S. subtype B Envs from the 1980s (N = 75) and 2010s (N = 785) (S4 Fig). The probability of each amino acid, indicated by one-letter symbols, at each position is indicated by the height of the letter. A gray bar indicates a gap or missing amino acid compared to the alignment, at that position. For PGDM1400, we can see a loss of Q at position 170. For 10–1074, we can see changes in potential N-linked glycosylation sites (PNGSs), including the N332 glycan. From our neutralization analysis, all PSVs from the 1980s had PNGS N332, while 15% of PSVs from our contemporary panel lacked the PNGS N332, making them resistant to NAb 10–1074 (S5 Fig). For VRC01, we can see variations across the contact sites, while the contact sites for 10E8 remain mostly unchanged. These results suggest that the accumulation of mismatched residues in individual NAb contact sites only partially contributes to the reduced neutralization sensitivity we observed for certain NAbs.

## Discussion

The diversity and mutability of HIV-1 remain as significant challenges for the development of effective vaccines and other products to prevent and treat HIV-1 [34]. Given the diversity in HIV-1 Env between subtypes, a median of 25% difference at the amino acid level [35], subtype-specific vaccines that are matched to regional circulating strains have been pursued. HIV-1 Envs from each subtype have similar antigenic features and neutralization-sensitive targets [36–38]. However, even within subtypes, a median Env amino acid difference of 15% exists [35]. Our study, along with others [4–7], demonstrates a continuing diversification of HIV-1 Env within subtype over time. Population level adaptation of HIV-1 has been shown to associate with reduced neutralization sensitivity and Env immunogenicity [4–7,24,25]. These adaptations may be impacted by humoral immune responses to natural infection, as well as partially effective vaccines and other products [39].

In this study, we focused on subtype B HIV-1 Env, as subtype B has been the primary driver of the HIV-1 epidemic in the United States (U.S.) for over forty years and is also a prominent subtype in North and South America, Europe, Australia, Middle East and North Africa. Bouvin-Pley et al. previously demonstrated reduced neutralization sensitivity of subtype B PSVs between 1987 and 2010 [6]. Significant decreases in sensitivity were observed for 8 out of the 13 NAbs tested, however no differences were observed in gp120 of V3 length or in number of PNGS [6]. In a further study, antigenic changes associated with reduced neutralization sensitivity also correlated with increased infectivity and reduced sensitivity to fusion inhibitors and CCR5 antagonists [40]. Similarly, increased diversification and reduced neutralization sensitivity have been reported for other subtypes. Rademeyer et al. showed that the subtype C epidemic in South Africa diversified significantly between 1998 and 2010 [25]. Diversification was not associated with changes in variable loop length or glycan density, however, significant decreases in neutralization sensitivity were observed for NAbs 4E10, VRC01 and PG9 [25]. Stefic et al. demonstrated a similar trend for the CRF02_AG epidemic in France between 1997 and 2012 [7]. For CRF02_AG, significant direct correlations between amino acid diversification and reduced neutralization sensitivity were observed for CD4bs NAbs 3BNC117 and NIH45-46.

Our analysis of U.S. subtype B HIV-1 encompassed a forty-year period and employed a larger number of NAbs that allowed us to identify trends associated with reduced Env neutralization sensitivity. We observed an association between the number of mismatched residues in the NAb contact site and reduced neutralization sensitivity. In some cases, this reduced sensitivity corresponded to loss of PNGS or other NAb contact site deviations, but not consistently. Amino acid divergence may also be associated with tertiary shifts in HIV-1 Env structure that could mitigate neutralization at the most sensitive Env targets. However, this study was not designed to evaluate this in depth.

Similar to the generalized amino acid divergence observed across Env, we observed reduced neutralization sensitivity of contemporary subtype B HIV-1 across Env domains over time. The degree of reduced neutralization sensitivity that we observed was associated with the potency of the specific NAb against early-pandemic HIV-1 strains. This supports the importance of bNAb potency in effective *in vivo* viral inhibition and in effecting population level changes in Env. Interestingly, while we observed a significant reduction in potency for NAbs with IC_50_s <0.5 μg/ml, we also observed a significant increase in potency for NAbs with IC_50_s >5 μg/ml. Env mutations that can circumvent potent NAbs may allow for changes in Env neutralization sensitivity as distal sites. As Env neutralization sensitivity can vary by HIV-1 subtype, patterns of reduced neutralization sensitivity will develop and vary accordingly. Most strikingly, subtype B and CRF01_AE HIV-1 have distinct neutralization profiles; while subtype B Envs are most sensitive to V3-specific NAbs, CRF01_AE Envs are most sensitive to V1V2-specific NAbs. Indeed, we observed the greatest decrease in NAb potency for V3-specific NAbs for subtype B and V1V2-specific NAbs for CRF01_AE. This trend of declining NAb potency was further demonstrated for three additional subtypes, using data from the LANL CATNAP database, as well as data from our laboratory. However, infection stage or neutralization sensitivity (tier) of the virus strains used in this meta-analysis were not factored into the data selection criteria, as the information was not available for many strains.

The recent AMP studies demonstrated that bNAbs could effectively prevent HIV-1 acquisition if the HIV-1 strain to which the individual is exposed is potently neutralized by the bNAb *in vitro* (IC_80_ [concentration of antibody that neutralizes 80% of the virus] <1 μg/ml). These results provide a baseline for effective prophylactic bNAb potency that was previously not defined [23]. The neutralization data generated in this study were produced using the same *in vitro* neutralization assay platform used to assess the AMP participant HIV-1 strains. Our results show that NAb potency of <1 μg/ml IC_80_ may be difficult to achieve against contemporary U.S. subtype B viruses. However, our NAb selection was based on availability at initiation of the study, and more potent reagents are currently in development or available. Only 5 of the 31 NAbs evaluated in this study achieved potency <1 μg/ml IC_80_ against more than 50% of the contemporary U.S. subtype B strains; only 3 NAbs (V3 specific PGT128 and 10–1074, and 10E8-iMAb) achieved a GM potency <1 μg/ml IC_80_ overall. 10E8-iMab potently neutralized the contemporary subtype B PSVs with a GM IC_80_ of 0.0139 μg/ml and 100% breadth. Wagh et al. previously demonstrated that the potency of 10E8-iMab exceeded <1 μg/ml IC_80_ against panels of subtype A, C, and D PSVs, with 96–100% neutralization coverage across the subtypes [41]. This antibody is now advancing in clinical trials; these types of antibodies are promising as they achieve the cross-subtype potency needed for globally effective products.

While reduced neutralization sensitivity over time was observed for HIV-1 Env, the changes reported in this study occurred over decades. We observed development of NAb resistance over time, as defined by the limits of our neutralization assay, but the contemporary Envs were not completely neutralization resistant. Additionally, this study only characterized HIV-1 Envs from a small convenience sample set from 30 individual infections. Changes in Env antigenicity may impact accessibility of contact sites for known bNAbs. However, these changes in antigenicity may also open opportunities for next generation bNAb targets on Env and development of novel bNAbs from contemporary infections should continue to be advanced. While changes in antigenicity over time may have reduced Env neutralization sensitivity, it is unknown how these changes have impacted other antibody functions, such as antibody-dependent cellular cytotoxicity (ADCC) and antibody-dependent cellular phagocytosis (ADCP), which are important parameters for additional study.

This work further highlights the need for continued surveillance of circulating HIV-1 strains and the inclusion of contemporary HIV-1 in vaccine analysis viral panels. As results from the AMP studies highlighted, the effectiveness of a given antibody-based treatment or vaccine is dependent on the sensitivity of the circulating Envs to the synthetic or elicited antibodies. Development and co-administration of next generation antibody-based prophylaxis may be required to achieve the level of breadth and potency needed to provide protective coverage [41]. Continued detailed characterization of circulating HIV-1 Envs should inform improved targeted development of immune-based interventions for the prevention and treatment of HIV-1.

## Materials and methods

### Cohort descriptions and ethics statement

Contemporary subtype B *envs* were cloned from plasma samples selected from the RV551 study, a non-human subjects research protocol approved by the Walter Reed Army Institute of Research (WRAIR) Human Subjects Protection Branch and the US Medical Research Development Command Office of Human Research Oversight. This study adhered to the policies for protection of human subjects, as prescribed in Army Regulations 70–25: Use of Volunteers as Subjects of Research. Formal consent was obtained in writing. RV551 was designed to assess the molecular virology and immunobiology of incident and prevalent contemporary U.S. Military HIV-1 subtype B infections. Samples were obtained from post residual HIV-1 test specimens submitted to the HIV Diagnostic and Reference Laboratory between 2017 and 2018 as part of universal screening for HIV-1 in U.S. military service members. Samples were anonymized and linked to HIV meta test data by a dis-interested data steward; Fiebig stage was determined by analysis of meta test data.

### HIV-1 Env sequencing

*Env* genes were sequenced via Single Genome Amplification (SGA). HIV-1 RNA was extracted from plasma using the QIAamp Viral RNA Mini Kit (Qiagen, Hilden, Germany). Complementary DNA (cDNA) was synthesized using Superscript III (Invitrogen Corp., Carlsbad, CA) as instructed by the manufacturer using the primer UNINEF-7’ (5’-GCACTCAAGGCAAGCTTTATTGAGGCTT-3’; HXB2 9632–9605 nt). Using near-endpoint dilution of cDNA template, a first round PCR was performed to amplify a near full length or 3’ half-length genome by using MSF12b/UNINEF-7’ (5’- GAAGCYATGCATGGACAAGTRGA-3’; 623–649 nt) or PolJv2/UNINEF-7’ (5’- GAAGCYATGCATGGACAAGTRGA-3’; HXB2 4371–4392 nt). From the first round PCR product, the *env* gene was amplified using the primers BH4minus (5’-TAGGCATTTCCTATGGCAGGAAGAAG; HXB2 5958–5983 nt) and BH2NOENZ2ACE (5’-GTCTCGAGATACTGCTCCTACTC; HXB2 8904–8882 nt). Both rounds of PCR were performed with Q5 polymerase (New England Biolabs, Ipswich, MA, USA). Amplicons were gel purified using the QIAquick Gel Extraction Kit (Qiagen, Hilden, Germany) and Sanger sequenced on a 3730xl capillary sequencher (Applied Biosystems Foster City, CA, USA). HIV-1 subtype was determined using the NCBI genotyping tool (https://www.ncbi.nlm.nih.gov/projects/genotyping/formpage.cgi).

### Phylogenetic and sequence analysis

For the phylogenetic analysis: RV551 amino acid sequences were aligned to 2011–2020 circulating subtype B Env sequences using the add function in the multiple sequence alignment program, MAFFT v7.475 [42]. The phylogeny was constructed from the alignment of subtype B and RV551 sequences using the IQ-TREE 2 software package for phylogenetic inference using the maximum likelihood (ML) criterion [43], with an HIV-1 between-patient matrix [44]. A sequence corresponding to the most recent common ancestor (MRCA) of subtype B Envs was reconstructed using FastML v.3.11 [PMID: 22661579] with branch-length optimization and a gamma distribution on subtype B Envs in the LANL database collected prior to 2010. The distribution of distances of RV551 sequences to the subtype B MRCA sequence was determined by computing pairwise distances to the consensus of sequences for each RV551 plasma sample assuming an HIV-1 between-sample matrix substitution model; the distribution of distances to the subtype B MRCA was also determined for subtype B Env sequences sampled between 1980–2010, between 2011–2020, in the U.S. between 2017–2018 and for the RV551 sequences. Pairwise distances were calculated across sequences within each subset. Shannon entropy, H = –∑*p_i_log*_2_*p_i_*, was calculated at each site for each subset. For the NAb contact site mismatch analysis, Env amino acid sequences for PSVs included in the neutralization analysis for each decade period were analyzed to determine the number of mismatched amino acids in the NAb contact sites. To visualize Env amino acid differences between decade periods, logo plots were generated using U.S. subtype B Envs selected from the LANL HIV sequence database from 1980s (N = 75) and 2010s (N = 785).

### Generation of envelope gene clones for PSV production

Envelope glycoprotein (gp160) genes were amplified by single genome amplification and cloned into eukaryotic expression vectors for use as PSVs, as previously described [45,46], Briefly, The amplicon was gel purified using the QIAquick Gel Extraction Kit (Qiagen, Hilden, Germany), cloned into pcDNA3.1/V5-His-TOPO eukaryotic cloning vector (Invitrogen, Waltham, MA, USA), and transformed into either STBL2 or STBL4 cells (Invitrogen, Waltham, MA, USA). Plasmid DNA was purified from bacterial cultures using the Qiagen Plasmid Maxi Kit (Qiagen, Hilden, Germany). Env sequence was confirmed by comparison with original SGA-derived amplicon. There were no amino acid differences between the cloned *env* genes and the uncloned *env* amplicon. Env function was confirmed by infection of the TZMbl reporter cell line (ATCC, Manassas, VA, USA).

### PSV production and titration

PSVs were prepared as previously described [46]. Briefly, PSVs were produced by co-transfection of HEK293T/17 cells (ATCC, Manassas, VA, USA) with a pSG3ΔEnv DNA plasmid encoding the HIV-1 backbone (24 μg DNA) and a plasmid encoding the HIV-1 *env* genes (8 μg DNA). Plasmids were combined with X-tremeGENE 9 DNA transfection reagent (Sigma-Aldrich, St. Louis, MO, USA) at a 1:3 ratio in serum-free DMEM, as per manufacturer recommendations. The transfection mix was added to a T75 flask containing plated HEK293T/17 cells in culture medium. The flask was incubated for 6 hours at 37°C. Then the media was replaced. PSVs were harvested 72 hours after transfection, filtered through a 0.45 μm PVDF membrane (MilliporeSigma, Burlington, MA, USA) and stored in 3 ml aliquots at -80°C. PSVs were then titrated on TZMbl cells to determine an acceptable dilution for neutralization assays. PSVs were serially diluted, then added to 384-well black-walled plates (Thermo Fisher Scientific, Waltham, MA, USA) in a final 12.5 μL/well volume. An additional 12.5 μL of supplemented DMEM was added to each well. TZM-bl cells were added to plates in 25 μL of supplemented DMEM with 40 μg/mL of DEAE-Dextran (Sigma, St. Louis, MO, USA). PSVs and cells were incubated together for 48 hours at 37°C. The supernatant was then removed from each well to leave a total of 25 μL of supernatant per well. Infectivity was measured by luminescence after adding 25 μL of the Bright-Glo Luciferase Assay System (Promega, Madison, WI, USA) to each well; luminescence was quantified using the EnVision luminometer (Perkin Elmer, Waltham, MA, USA).

### Coreceptor determination by the GHOST cell assay

The GHOST cell infection assay was used to determine PSV coreceptor usage. Parental, CXCR4-expressing, or CCR5-expressing GHOST cells were cultured in 96-well plates at 1.6x10^4^ cells/well and incubated at 37°C overnight. Cells were infected with undiluted PSV in the presence of 20 μg/ml of polybrene infection reagent (MilliporeSigma, Billerica, MA, USA) for 4 hours. Cells were washed and cultured for 2 days, then harvested and fixed with 4% paraformaldehyde. The percentage of infected cells expressing green fluorescent protein was measured by flow cytometry analysis using an LSRII cytometer and FACSDIVA software (Becton-Dickinson, San Jose, CA, USA). Post-acquisition analysis was conducted with FlowJo software (FlowJo, LLC, Ashland, OR, USA). PSVs were designated as using the CXCR4 or CCR5 receptor if the ratio to control negative (RTCN) was >10; RTCN = (MFI x %pos)_infected_ / (MFI x %pos)_uninfected_. Assay controls included an uninfected negative control, murine leukemia virus as a positive control, and known CCR5 and CXCR4 utilizing PSVs as cell line controls.

### Antibodies and plasma

The following reagents were obtained through the HIV Reagent Program, Division of AIDS, NIAID, NIH (Manassas, VA, USA): 10–1074, 10E8, 2191, 2219, 3074, 35O22, 3869, 3BNC117, 7H6, CH02, CH03, CH04, HJ16, NIH45-64, PGT121, PGT126, PGT128, PGT145, sCD4, VRC01, and VRC03. The following reagents were purchased from Polymun (Klosterneuburg, Austria): 2F5, 2G12, 447-52D, 4E10, b12, PG16, PG9. VRC07 and 10E8-iMab were graciously provided by Yaoxing Huang and David Ho from the Aaron Diamond AIDS Research Center, Columbia University Medical Center. PGDM1400 and PGT130 were graciously provided by Dr. Devin Sok at the International AIDS Vaccine Initiative. 830A was graciously provided by Dr. Susan Zolla-Pazner at the Icahn School of Medicine at Mount Sinai, New York, NY.

Additional samples for longitudinal virus characterization were also obtained from the U.S. Military HIV Natural History Study (NHS), a 37-year prospective, continuous enrollment cohort study of active duty service members and Military Health System beneficiaries infected with HIV [47]. The NHS protocol has been IRB-approved centrally at the Uniformed Services University of the Health Sciences, with review at participating sites and WRAIR. All NHS subjects provide written informed consent. All selected plasma samples were from chronic, subtype B HIV infections.

### PSV neutralization

Neutralizing antibody titers were determined using TZM-bl cells [48] in a high-throughput assay utilizing robotic liquid handling, as previously described [33]. Plasma samples were prepared by heat inactivation at 56°C for 45 min, followed by 6 min centrifugation at 3000 rpm to remove particulates. Plasma samples were prepared at a starting dilution of 1:20 in supplemented DMEM; NAbs were prepared at a starting concentration of 25 μg/mL in supplemented DMEM. PSVs were diluted to a concentration to yield 100,000 relative light units (RLUs) and at least 3 times above the cell-only controls, as determined by the PSV titration assay. Neutralization reagents were serially diluted using a Biomek NXP liquid handler (Beckman Coulter, Indianapolis, IN, USA). Titered NAbs and plasma samples were transferred to 384-well culture plates and incubated with an equal volume of PSV for 1 hour at 37°C. TZMbl cells (3x10^3^ cell/well) mixed with 40 μg/mL DEAE-dextran (Sigma, St. Louis, MO, USA) were added to each well and incubated for an additional 48 hours. RLUs were detected with the EnVision luminometer (Perkin Elmer, Waltham, MA, USA) using the Bright-Glo Luciferase Assay System (Promega Corporation, Madison, WI, USA). Percentage neutralization (percentage reduction of relative light units in the presence of plasma) was calculated for each plasma dilution. Neutralization dose–response curves were fitted by nonlinear regression using the LabKey Server, and the final titer is reported as the concentration of NAb (IC_50_/IC_80_) or reciprocal of the dilution of plasma (ID_50_/ID_80_) necessary to achieve 50% or 80% neutralization.

### Meta-data selection

Neutralization data for historic subtype B, and non-subtype B PSVs were selected from the CATNAP Server on the LANL HIV database. For all meta-data analysis, we utilized data from all HIV strains that matched our decade, location, and subtype selection criteria; HIV strains were removed if they were known to utilize the CXCR4 coreceptor. The number of HIV strains included in all meta-data analyses are shown in S1 Table.

### Statistical analysis

Significant differences between neutralization titers were determined by Mann Whitney U test or Wilcoxon matched-pairs signed rank, as indicated; * = p<0.01, ** = p<0.001, *** = p<0.0001. The Jonckheere’s trend test was used to determine statistically significant trends over time. Significant correlations between data sets were determined by Spearman correlation; where relevant, trend lines were shown.

## Supporting information

S1 Fig Neutralization sensitivity of early-stage versus late-stage subtype B HIV-1 Envs. Pseudoviruses were categorized by infection stage to demonstrate differences in neutralization sensitivity between Envs from early-stage infections (Fiebig I-IV, black bars), as compared to late-stage infections (Fiebig V-VI, red bars). The red dotted line at 50 indicates the negative cutoff value for this assay. Significant differences were determined by Mann Whitney U test; * = p<0.01.
(TIFF)

S2 Fig Correlation between neutralization endpoints. Neutralization endpoints were compared between data generated in our MHRP (U.S. Military HIV Research Program) laboratory and data available on the CATNAP (Compile, Analyze and Tally Nab Panels) Server on the Los Alamos National Laboratory (LANL) HIV database. Neutralization log_10_ IC_50_ values were compared for the same 3 pseudoviruses, including SF162, MN and AC10, against 18 NAbs. The correlation between data sets was determined by Spearman correlation; the trend line is shown.
(TIFF)

S3 Fig Changes in HIV+ plasma neutralization sensitivities of subtype B HIV-1 Envs over time. Neutralization potencies of 33 subtype B HIV+ plasma samples collected in the 1990s, 2000s and 2010s were determined against subtype B pseudoviruses (PSVs) with Envs isolated in the 1980s, 1990s, 2000s and 2010s. The A) geometric mean (GM) score of plasma ID_50_ values and B) breadth (% of neutralized viruses) for each plasma:virus decade period were determined. C) The heat map shows the log10 ID_50_ value of each plasma sample against each PSV. SF162 PSV, determined to be exceptionally neutralization-sensitive, was excluded from the analysis but the data are included here for reference.
(TIFF)

S4 Fig Broadly neutralizing antibody contact sites for 1980s and 2010s HIV-1 Envs. Subtype B HIV-1 Env sequences from the 1980s (N = 75) and 2010s (N = 785) were from the Los Alamos National Laboratory HIV sequence database. Amino acid contact sites of four broadly neutralizing antibodies, including PGDM1400 (top left), 10–1074 (top middle), 10E8 (top right), and VRC01 (bottom), are shown as logo plots for each decade period. The probability of each amino acid, indicated by one-letter symbols, at each position is indicated by the height of the letter. A gray bar indicates a gap, or missing amino acid compared to the alignment, at that position.
(TIFF)

S5 Fig Impact of N332 glycan on 10–1074 broadly neutralizing antibody neutralization. Pseudoviruses from the 1980s and 2017–18 periods were evaluated for presence (N332+) or absence (N332-) of asparagine at Env position 332. Env sensitivity to the 10–1074 broadly neutralizing antibody is demonstrated for N332+ and N332- pseudoviruses. NA indicates that no Envs of that genotype were available. Significant differences were determined by Mann Whitney U test; *** = p<0.0001.
(TIFF)

S1 Table Number of viruses utilized from each decade for mAb neutralization analyses. The number of pseudoviruses tested from each decade using each NAb are tabulated for the analyses presented in the indicated figures.
(TIFF)
