# Supplementary figures and images for: Evolution of HIV-1 envelope towards reduced neutralization sensitivity, as demonstrated by contemporary HIV-1 subtype B from the United States
Source: PLoS Pathog. 2023 Dec 6;19(12):e1011780. doi: 10.1371/journal.ppat.1011780 (PMC10727358; doi:10.1371/journal.ppat.1011780)

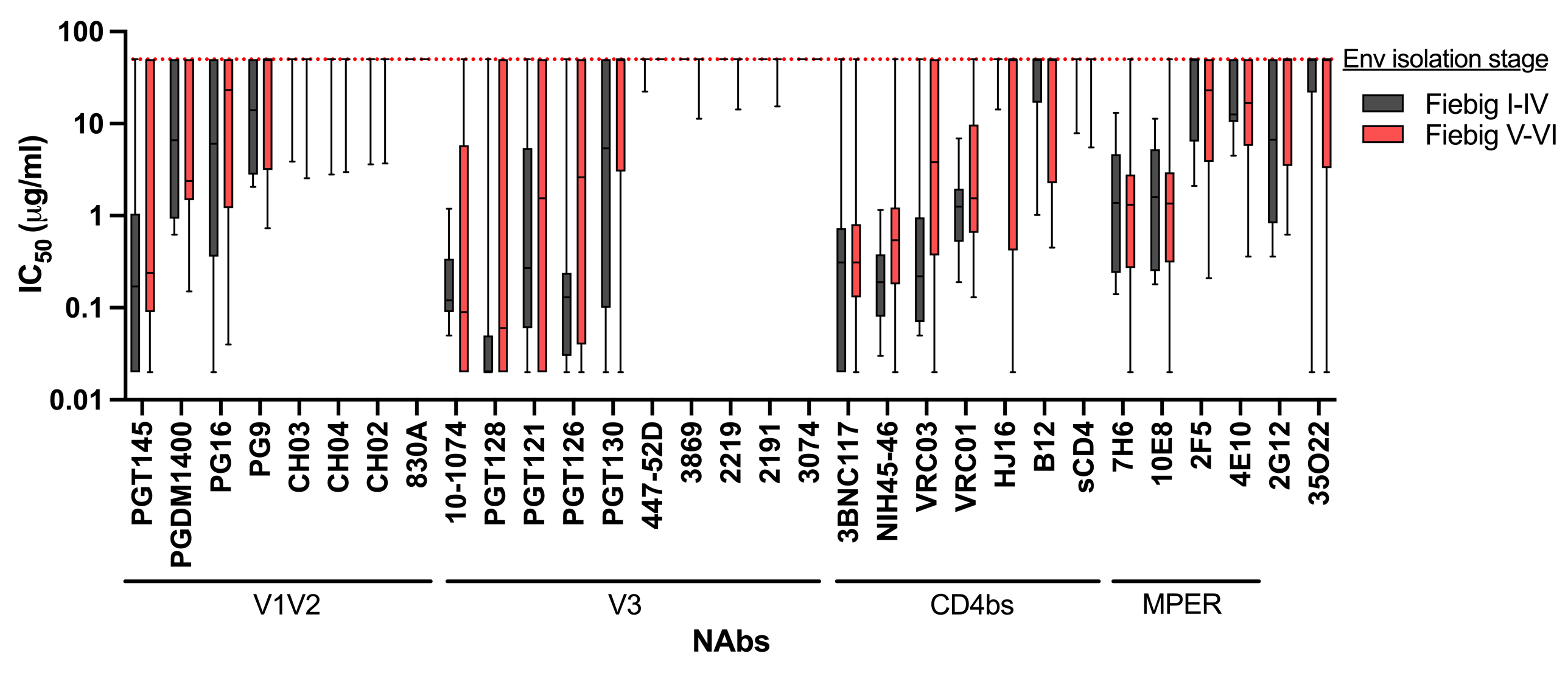

Supplement: S1 Fig — Pseudoviruses were categorized by infection stage to demonstrate differences in neutralization sensitivity between Envs from early-stage infections (Fiebig I-IV, black bars), as compared to late-stage infections (Fiebig V-VI, red bars). The red dotted line at 50 indicates the negative cutoff value for this assay. Significant differences were determined by Mann Whitney U test; * = p<0.01. (TIFF) [file ppat.1011780.s001.tiff]

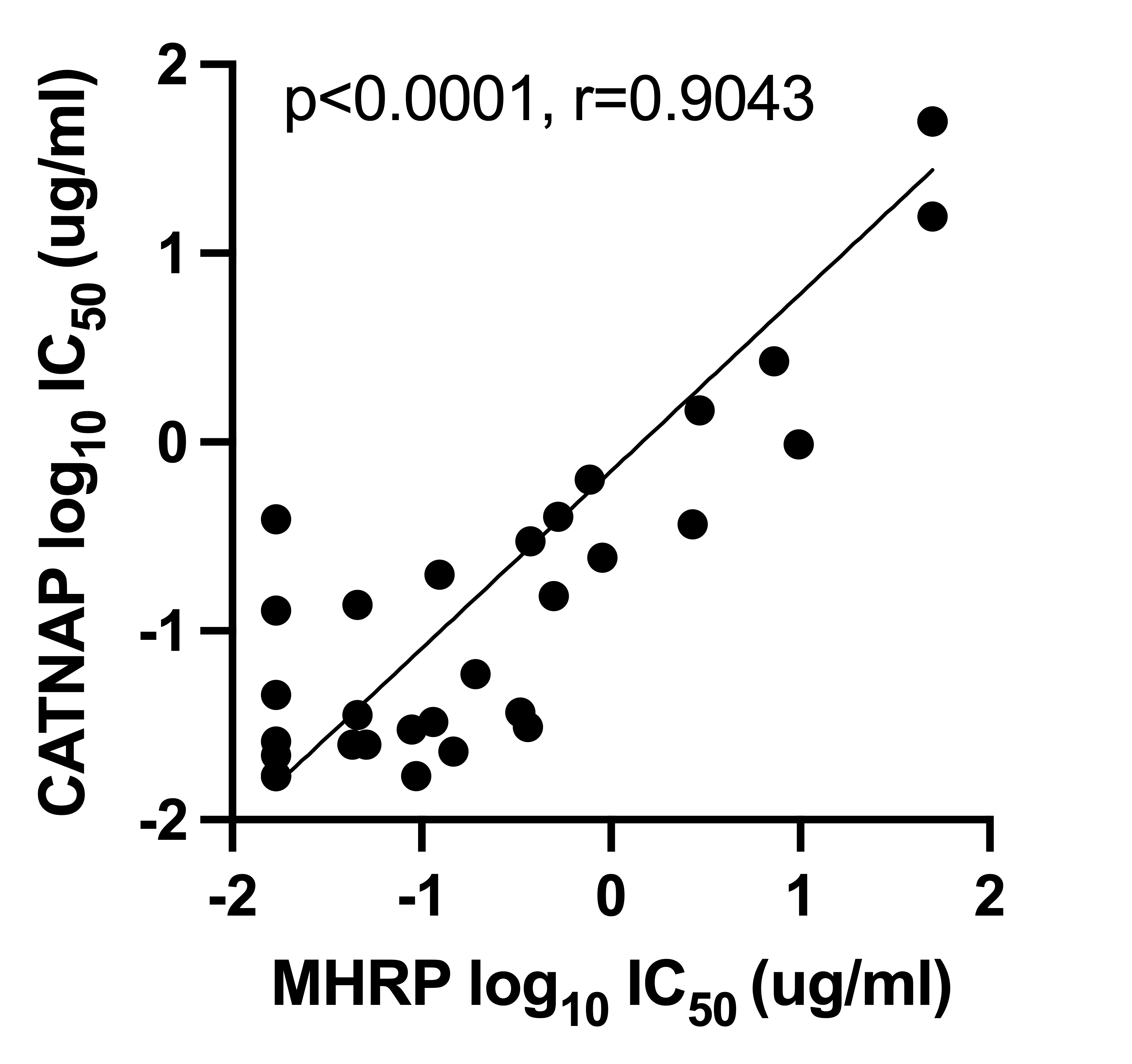

Supplement: S2 Fig — Neutralization endpoints were compared between data generated in our MHRP (U.S. Military HIV Research Program) laboratory and data available on the CATNAP (Compile, Analyze and Tally Nab Panels) Server on the Los Alamos National Laboratory (LANL) HIV database. Neutralization log10 IC50 values were compared for the same 3 pseudoviruses, including SF162, MN and AC10, against 18 NAbs. The correlation between data sets was determined by Spearman correlation; the trend line is shown. (TIFF) [file ppat.1011780.s002.tiff]

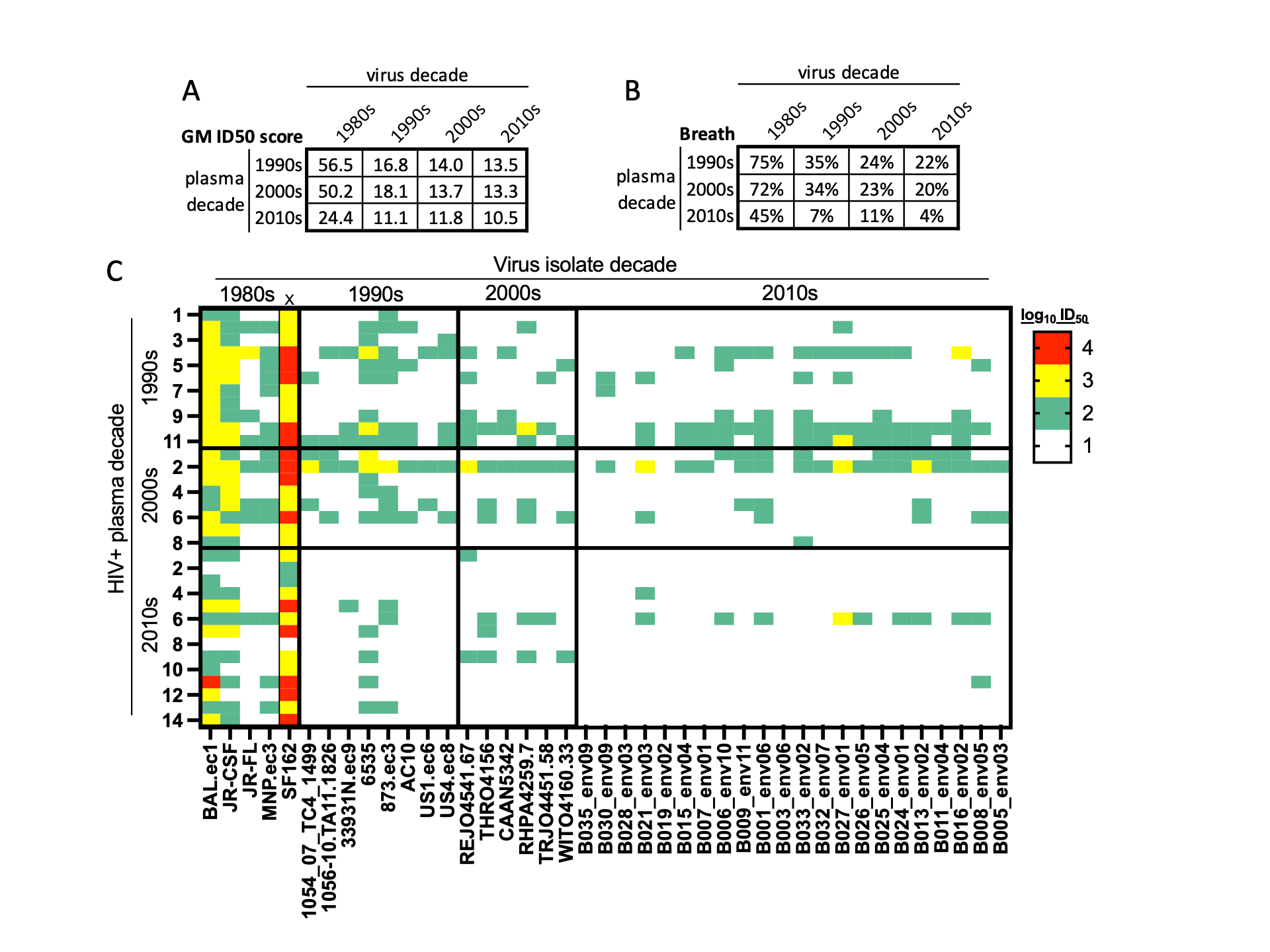

Supplement: S3 Fig — Neutralization potencies of 33 subtype B HIV+ plasma samples collected in the 1990s, 2000s and 2010s were determined against subtype B pseudoviruses (PSVs) with Envs isolated in the 1980s, 1990s, 2000s and 2010s. The A) geometric mean (GM) score of plasma ID50 values and B) breadth (% of neutralized viruses) for each plasma:virus decade period were determined. C) The heat map shows the log10 ID50 value of each plasma sample against each PSV. SF162 PSV, determined to be exceptionally neutralization-sensitive, was excluded from the analysis but the data are included here for reference. (TIFF) [file ppat.1011780.s003.tiff]

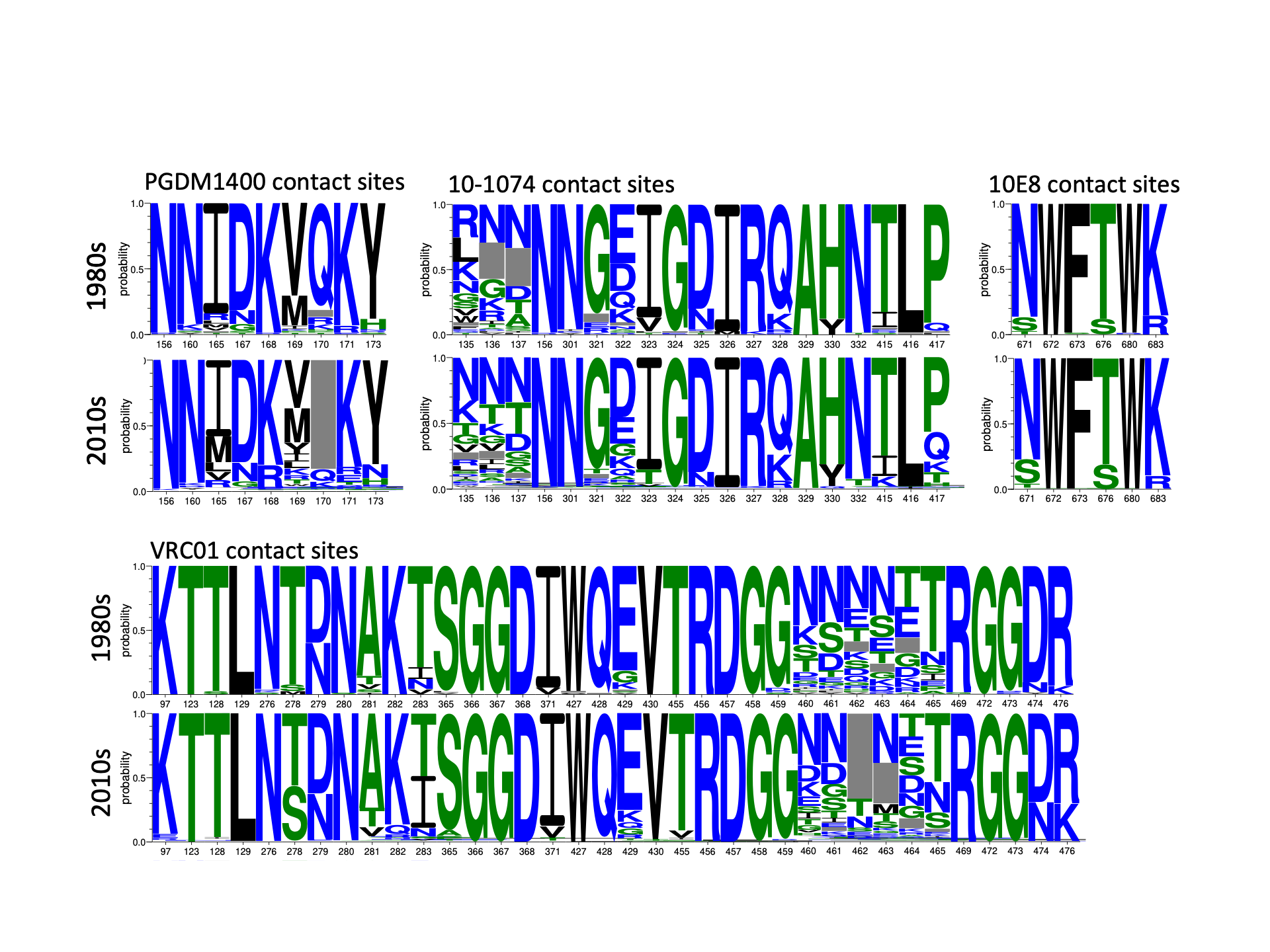

Supplement: S4 Fig — Subtype B HIV-1 Env sequences from the 1980s (N = 75) and 2010s (N = 785) were from the Los Alamos National Laboratory HIV sequence database. Amino acid contact sites of four broadly neutralizing antibodies, including PGDM1400 (top left), 10–1074 (top middle), 10E8 (top right), and VRC01 (bottom), are shown as logo plots for each decade period. The probability of each amino acid, indicated by one-letter symbols, at each position is indicated by the height of the letter. A gray bar indicates a gap, or missing amino acid compared to the alignment, at that position. (TIFF) [file ppat.1011780.s004.tiff]

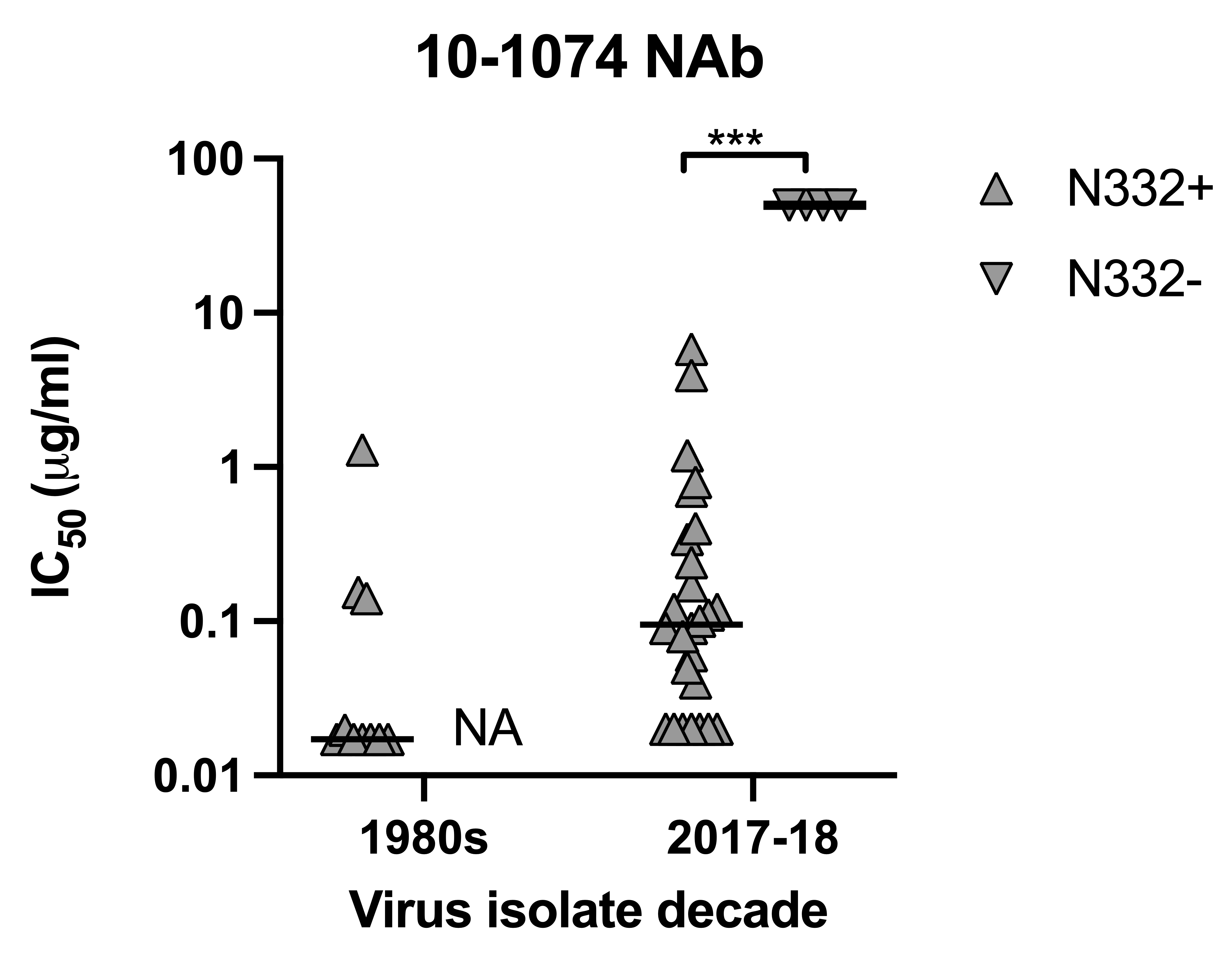

Supplement: S5 Fig — Pseudoviruses from the 1980s and 2017–18 periods were evaluated for presence (N332+) or absence (N332-) of asparagine at Env position 332. Env sensitivity to the 10–1074 broadly neutralizing antibody is demonstrated for N332+ and N332- pseudoviruses. NA indicates that no Envs of that genotype were available. Significant differences were determined by Mann Whitney U test; *** = p<0.0001. (TIFF) [file ppat.1011780.s005.tiff]

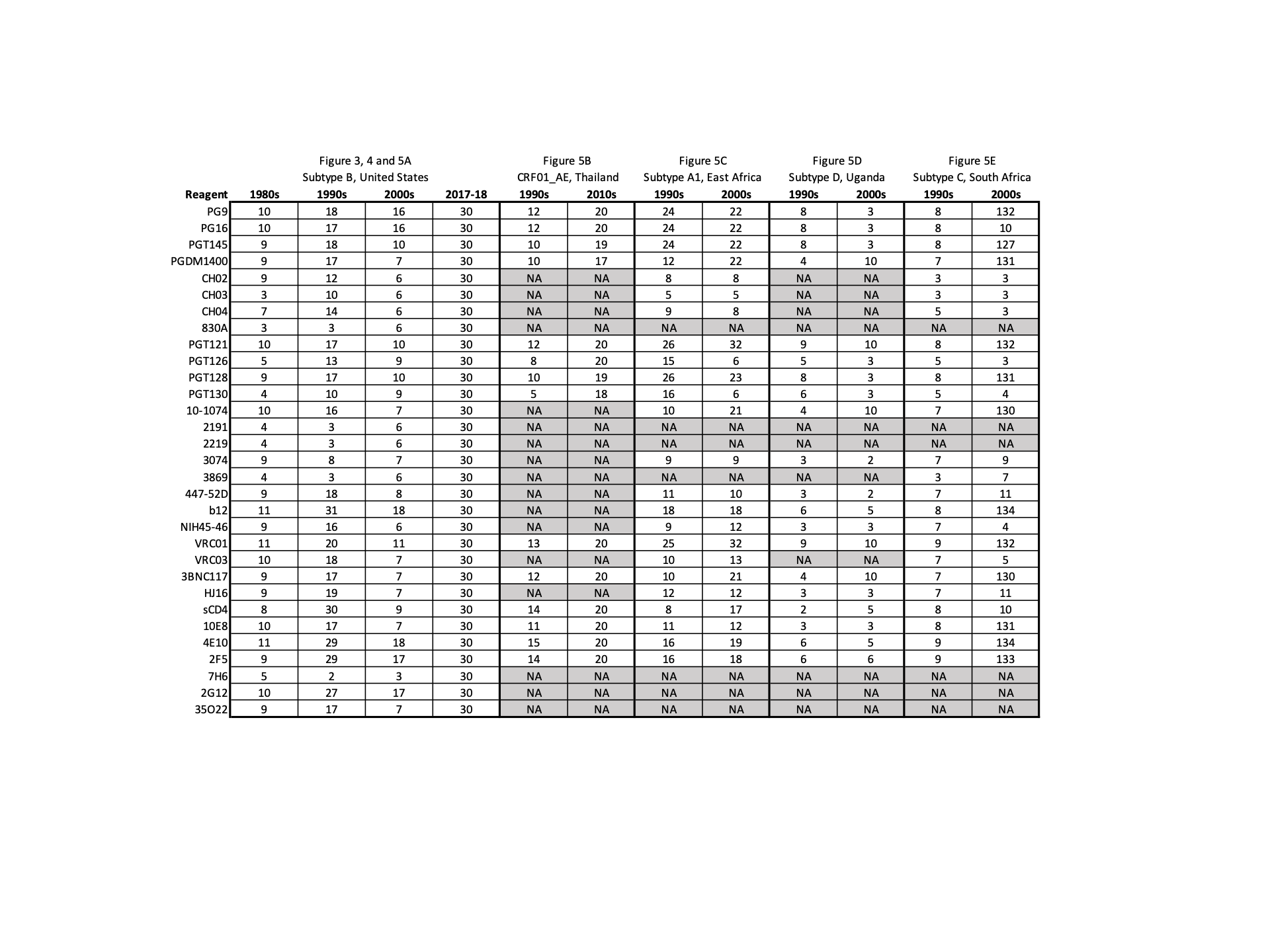

Supplement: S1 Table — The number of pseudoviruses tested from each decade using each NAb are tabulated for the analyses presented in the indicated figures. (TIFF) [file ppat.1011780.s006.tiff]
